# Supplementary material for: Novel Input for Designing Patient-Tailored Pulmonary Rehabilitation: Telemonitoring Physical Activity as a Vital Sign—SMARTREAB Study
Source: J Clin Med. 2020 Jul 31;9(8):2450. doi: 10.3390/jcm9082450 (PMC7464888; doi:10.3390/jcm9082450)
Supplement: Supplementary file 1 [file jcm-09-02450-s001.pdf]

Supplementary materials

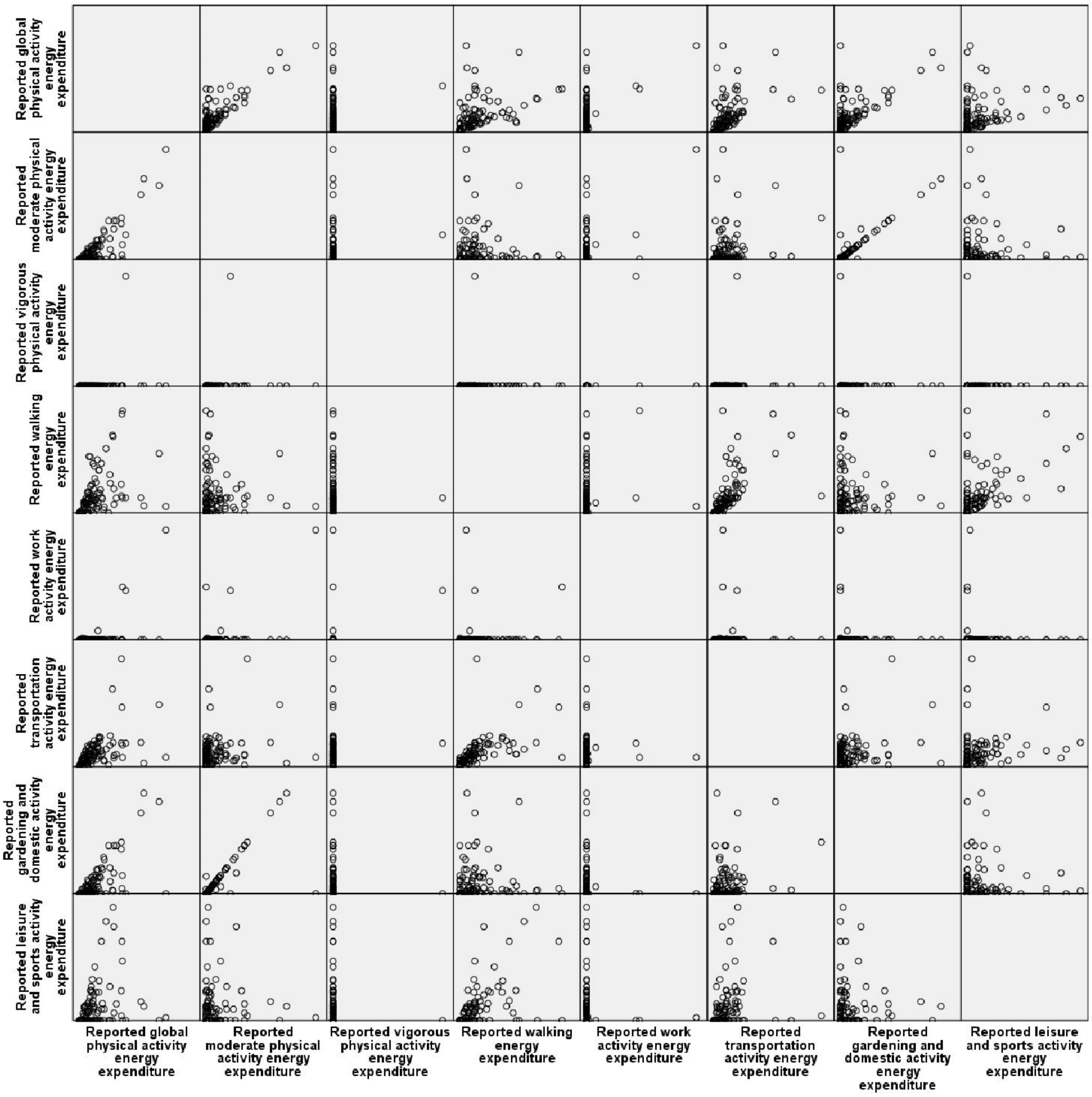

Figure S1. Pair-wise scatter plots in a correlations matrix format, showing the International Physical Activity Questionnaire (IPAQ) total score association with its component subscores.

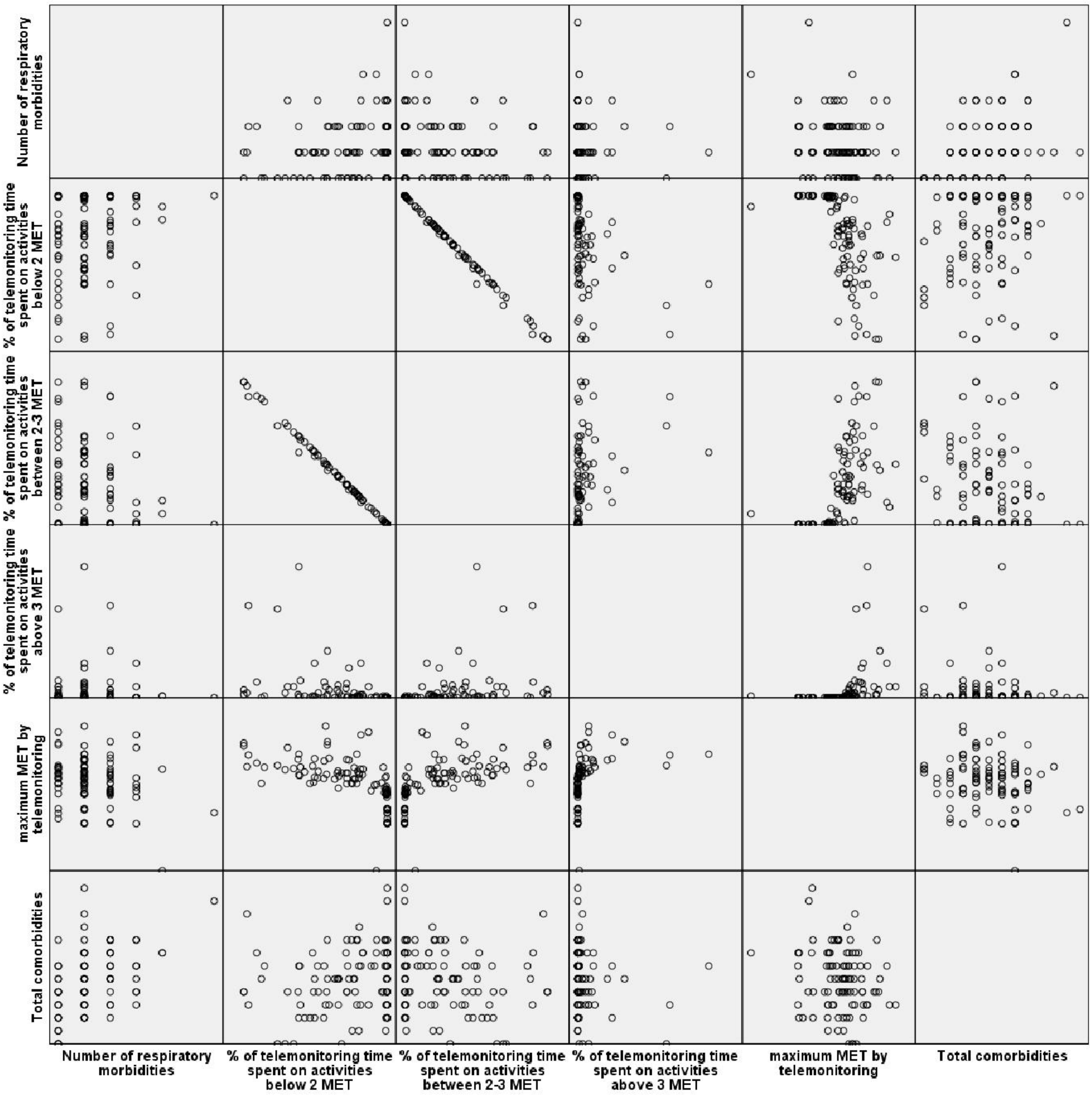

Figure S2. Pair-wise scatter plots in a correlations matrix format, showing the association between physical activity telemonitoring results and total number of comorbidities or respiratory comorbidities.

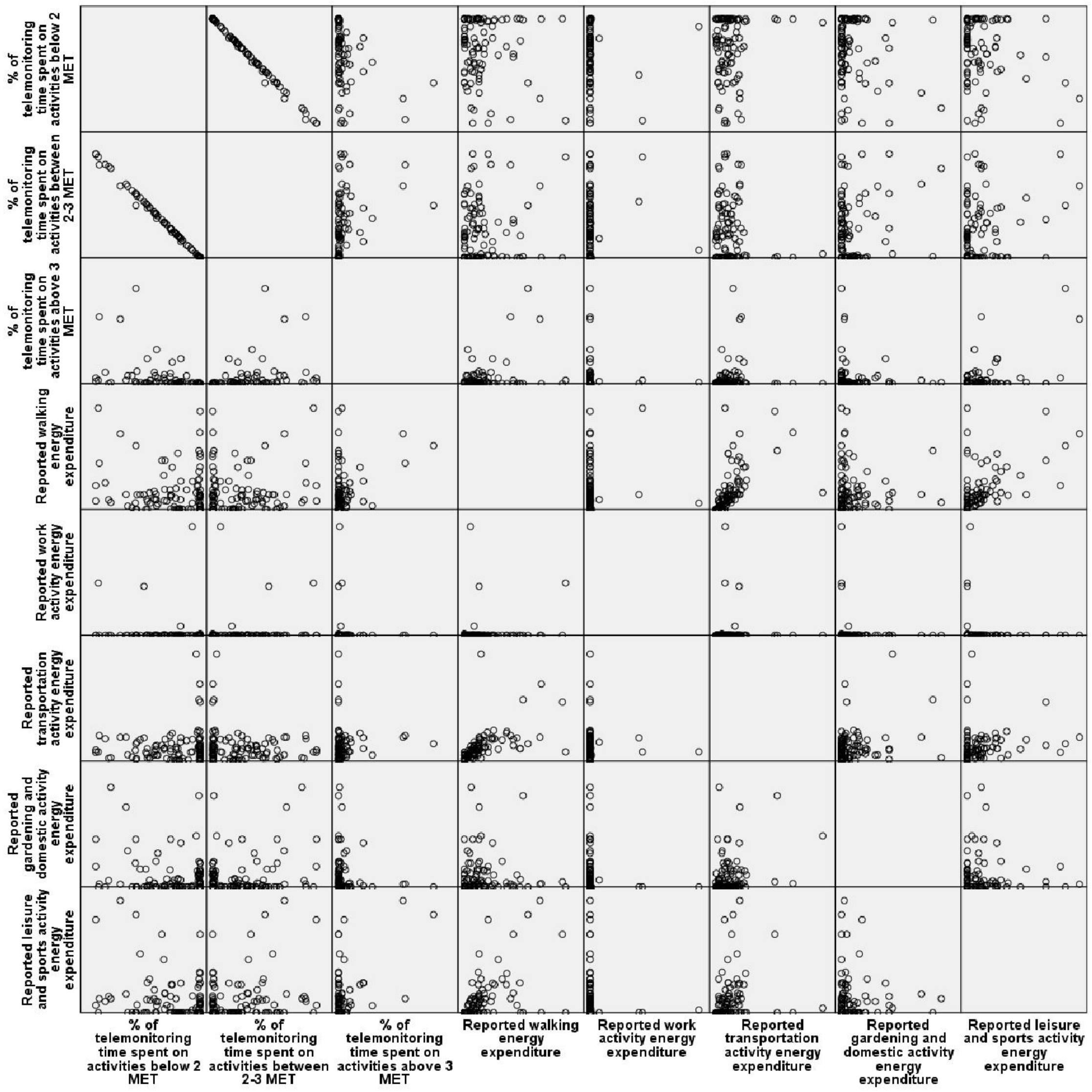

Figure S3. Pair-wise scatter plots in a correlations matrix format, showing the association between telemonitoring results of time spent on activities below 2 METs, between 2 and 3 METS, and above 3 METS with International Physical Activity Questionnaire (IPAQ) subscores.

Table S1 – Subjective and objective PADL assessments per chronic respiratory disease

	**Total**	**COPD**	**ILD**	**Asthma**	**Bronch.**	**Others**
	n = 100	*n* = 41	*n* = 22	*n* = 15	*n* = 10	*n* = 12
	**SMARTREAB Telemonitoring *PADL < 2 METs* (% time; mean ± SD)**					
**IPAQ categories**						
Low (*n* = 20)	83.2 ± 14.5	80.4 ± 16.1	89.3 ± 10.7	0.0 ± 0.0	82.7 ± 18.8	85.4 ± 0.0
Moderate (*n* = 49)	83.0 ± 19.2	84.0 ± 19.7	84.6 ± 18.6	86.3 ± 16.0	91.4 ± 12.0	66.6 ± 23.9
High (*n* = 31)	73.4 ± 25.8	75.6 ± 28.1	74.1 ± 27.9	75.7 ± 27.7	84.8 ± 22.5	56.6 ± 20.1
	**SMARTREAB Telemonitoring *PADL from 2 to 3 METs* (% time; mean ± SD)**					
**IPAQ categories**						
Low (*n* = 20)	16.6 ± 14.4	19.4 ± 15.9	10.5 ± 10.4	0.0 ± 0.0	17.2 ± 18.8	14.6 ± 0.0
Moderate (*n* = 49)	16.6 ± 18.7	15.6 ± 18.8	15.3 ± 18.6	13.5 ± 15.7	8.7 ± 12.0	32.5 ± 23.7
High (*n* = 31)	25.9 ± 25.4	23.5 ± 27.1	25.5 ± 28.0	24.2 ± 27.5	15.1 ± 22.4	41.5 ± 20.7
	**SMARTREAB Telemonitoring *PADL > 3 METs* (% time; mean ± SD)**					
IPAQ categories						
Low (n=20)	0.2 ± 0.3	0.2 ± 0.3	0.2 ± 0.3	0.0 ± 0.0	0.1 ± 0.1	0.0 ± 0.0
Moderate (n=49)	0.4 ± 1.0	0.5 ± 1.3	0.1 ± 0.2	0.3 ± 0.5	0.0 ± 0.0	0.9 ± 1.3
High (n=31)	0.7 ± 1.8	0.8 ± 2.0	0.4 ± 0.8	0.1 ± 0.1	0.1 ± 0.1	1.9 ± 3.3

COPD: chronic obstructive pulmonary disease; ILD: interstitial lung disease; Bronch.: bronchiectasis; Others: post-thoracic surgery, lung cancer, tuberculosis sequelae, lung disorder associated with connective tissue disease and pulmonary ossification; PADL: physical activity in daily life; MET: metabolic equivalence of task; IPAQ: international physical activity questionnaire; SD: standard deviation.
